# Racial Disparities in Inpatient Hospital Outcomes of Primary Sclerosing Cholangitis in United States: Nationwide Analysis

**DOI:** 10.3390/diagnostics14222493

**Published:** 2024-11-07

**Authors:** Ishaan Vohra, Harishankar Gopakumar, Dushyant Singh Dahiya, Michel Kahaleh, Neil Sharma

**Affiliations:** 1 Department of Gastroenterology and Hepatology, University of Illinois College of Medicine at Peoria, 530 NE Glen Oak Ave, Peoria, IL 61637, USA; hgopakum@uic.edu; 2 Division of Gastroenterology, Hepatology & Motility, The University of Kansas School of Medicine, Kansas City, KS 66045, USA; ddahiya@kumc.edu; 3 Foundation of Interventional and Therapeutic Endoscopy, New Brunswick, NJ 07103, USA; mkahaleh@gmail.com; 4 Division of Gastroenterology, Rutgers The State University of New Jersey, New Brunswick, NJ 07103, USA; 5 Parkview Cancer Institute, Advanced Interventional Endoscopy & Endoscopic Oncology (IOSE) Division, GI Oncology Program, 11104 Parkview Circle, Suite 310, Fort Wayne, IN 46845, USA; nrsharma219@yahoo.com

**Keywords:** cholangitis, nationwide analysis, mortality, cholangiocarcinoma, hepatocellular carcinoma, gall bladder cancer, cirrhosis, Charlson Comorbidity Index, liver transplant, varices, encephalopathy, portal hypertension

## Abstract

**Background:** Primary sclerosing cholangitis (PSC) is an idiopathic cholestatic liver disease that may lead to biliary strictures and destruction. It is associated with p-ANCA positivity and inflammatory bowel disease, typically ulcerative colitis. The aim of this study is to investigate the trends of inpatient healthcare utilization and mortality from 2008 to 2017 in the United States. **Methods:** The Nationwide Inpatient Sample (NIS) was examined to identify adult patients diagnosed with PSC between 2008 and 2017. Data on patient demographics, resource utilization, mortality, and PSC-related complications were collected. STATA version 16.0 was employed to perform forward stepwise multivariate regression analysis, generating adjusted odds ratios for both primary and secondary outcomes. Primary outcomes included the inpatient mortality rate and healthcare resource utilization (length of stay, total charges, and trends over the study period). Secondary outcomes focused on trends in associated comorbidities and malignancies in patients with PSC. **Results:** The average total charge increased by 32.2% ± 2.12 from USD 61,873 ± 2567 in 2008 to USD 91,262 ± 2961 in 2017. Concurrently, the average length of stay declined from 8.07 ± 0.18 days in 2008 to 7.27 ± 0.13 days in 2017. The APR-DRG severity of illness and risk of death significantly increased (major or extreme) during the study period (2008 to 2017), with severity rising from 73.6% to 82.7% (coefficient: 0.21, 95% CI: 0.13–0.28) and risk of death from 45.3% to 60.9% (coefficient: 0.15, 95% CI: 0.08–0.23). The proportion of patients with HCC increased from 1.3% to 7.9% (coefficient: 2.13, 95% CI: 1.9–2.8). Conversely, the percentage of patients with cholangiocarcinoma (CCA) decreased from 5.1% to 2.8% (coefficient: −0.36, 95% CI: −0.25 to −0.46). **Conclusions:** There was rising mortality and healthcare resource utilization among patients with PSC from the years 2008 to 2017. These trends were paralleled by increasing rates of decompensated cirrhosis, HCC, and liver transplants. However, the incidence of CCA decreased during this time period. African American patients with PSC had worse inpatient mortality outcomes and healthcare utilization as compared to white patients. Further studies are warranted to investigate a possible causal link amongst these trends.

## 1. Introduction

Primary sclerosing cholangitis (PSC) is a chronic and idiopathic cholestatic liver disease, where inflammation and fibrosis can lead to multifocal extrahepatic and intrahepatic biliary strictures and destruction [1]. The pathogenesis of this immune-mediated disease remains unclear, but both genetic and environmental factors are believed to play a role. The diagnosis of PSC requires the exclusion of secondary causes of cholangitis such as acquired immunodeficiency syndrome (AIDS)-related cholangiopathy, choledocholithiasis, amyloidosis, sarcoidosis, ischemic cholangiopathy, and IgG4-associated disease. Perinuclear anti-neutrophil cytoplasmic antibodies (p-ANCAs) are found in 26 to 85% of PSC patients [2]. PSC is more common in men (approximately two-thirds of cases) and in patients with pancolitis [3]. The median age of diagnosis is 41 years old [4]. Up to 60–80% of patients with PSC have underlying inflammatory bowel disease, usually ulcerative colitis [5]. In patients with inflammatory bowel disease (IBD), activated lymphocytes from the inflamed gut enter the enterohepatic circulation and cause hepatic inflammation. Additionally, dysbiosis of the gut microbiome and increased intestinal permeability can lead to the translocation of bacterial metabolites from the inflamed gut to the liver [5]. In addition to IBD, PSC has been associated with pancreaticobiliary malignancy, gall bladder malignancy, autoimmune hepatitis overlap syndrome, and colon cancer. PSC may initially present asymptomatically with a cholestatic liver enzyme elevation [6]. However, of those symptomatic patients, abdominal pain, fatigue, and pruritus are most commonly observed. Its prognosis is poor, with most cases resulting in end-stage liver disease requiring liver transplantation. No medical therapy has been shown to alleviate the disease course of PSC. Its estimated incidence is from 1 to 2 per 10,000 people annually in the European population, with the average ranges of its incidence and prevalence per 10,000 varying from 0.3 to 5.8 and from 1.9 to 40.2, respectively [3]. Studies in northern Europe showed a rising prevalence of PSC [7,8]. However, in the United States, the trends in the prevalence and mortality of PSC are not well understood. The aim of this study is to perform multi-center, population-based research on the nationwide trends of PSC hospital admissions, healthcare burden, and associated risk factors over ten years (from 2008–2017) by querying the Nationwide Inpatient Sample (NIS) database.

## 2. Methods

### 2.1. Data Source

This study utilizes nationwide data, utilizing the Healthcare Cost and Utilization Project (HCUP) Nationwide Inpatient Sample (NIS) database provided by the Agency for Healthcare Research and Quality. Covering the years from 2008 to 2017, the NIS is recognized as the most extensive publicly accessible all-payer inpatient healthcare database in the United States [9,10]. The NIS database has grown significantly over time; by 2017, it included data from 35 million (weighted) hospital stays across 4584 hospitals in 48 states within the United States. This study utilizes the most recent NIS databases available, specifically from the years 2016 and 2017 [11].

The Nationwide Inpatient Sample (NIS) is structured as a stratified probability sample that accurately represents all non-federal acute care inpatient hospitalizations across the country. Hospitals are categorized by bed count, geographic region, teaching status, urban or rural designation, and ownership/control. From each stratum, a 20% probability sample is selected. The NIS encompasses all hospital discharges, documenting patient details such as primary and secondary diagnoses, demographics, and discharge status. The dataset features unique identifiers, demographic variables, discharge dispositions, primary and secondary diagnoses (up to 40), primary procedure codes (up to 25), hospital characteristics, and nearly 100 variables related to patients and hospitals. To ensure national representation, each discharge is weighted based on the ratio of the total number of discharges from all acute care hospitals in the United States to the number of discharges in the 20% sample.

The Nationwide Inpatient Sample (NIS) database covers about 97% of the US population and aligns with the National Hospital Discharge Survey, ensuring the reliability of its data [12]. The Healthcare Cost and Utilization Project (HCUP) has implemented quality control procedures that confirm the accuracy of the principal diagnoses and hospitalization dates [13]. Thus, this database is a reliable source for representing patients admitted with primary sclerosing cholangitis in the United States. The NIS contains both patient-level and hospital-level information, recording up to 40 discharge diagnoses and 25 procedures per patient using the International Classification of Diseases, Ninth Revision, Clinical Modification (ICD-9-CM) and the Tenth Revision, Clinical Modification (ICD-10-CM).

### 2.2. Study Population

We queried the NIS using the ICD-9-CM (576.1) and ICD-10-CM (K83.0 and K83.01) diagnosis codes to identify all admissions for patients with primary sclerosing cholangitis (PSC). Patients under 18 years of age were excluded. The specific ICD-9-CM/ICD-10-CM diagnostic and procedural codes employed in this study are detailed in the Supplementary Materials (Supporting Table S1). We collected data on patient and hospital characteristics, illness severity, demographics, and resource utilization. Additionally, we gathered information on associated malignancies, such as hepatocellular carcinoma (HCC), cholangiocarcinoma (CCA), colorectal cancer (CRC), and gallbladder cancer (GB). Complications including secondary portal hypertension, cholangitis, common bile duct stones, ascites, hepatic encephalopathy, and gastroesophageal varices were identified using the ICD-9 and ICD-10 codes (Supporting Table S1). This study did not require institutional review board approval due to the de-identified nature of the NIS database and its public availability.

### 2.3. Study Variables

The considered patient-level variables were age, sex, and race (categorized as Caucasian, African American, Hispanic, and others, including Asian and Native American). We also considered the primary expected payer (Medicare, Medicaid, private insurance, and uninsured), hospital bed size (small, medium, and large), teaching status, hospital region (Northeast, Midwest, South, and West), and urban location. The comorbidity burden for risk adjustment was assessed using the Agency for Healthcare Research and Quality (AHRQ) comorbidity measures, based on the Deyo adaptation of the Charlson Comorbidity Index (CCI) for administrative data [14]. Additionally, we utilized the All Patient Refined Diagnosis Related Group (APR-DRG) system to estimate the severity of illness, risk of mortality, prognosis, treatment difficulty, and resource intensity, which has been validated for its clinical utility and resource utilization [15].

The mortality rate, patient discharge status, hospital length of stay (LoS), total charges (the amount billed by the hospital for services rendered), and actual cost of care (adjusted for inflation to 2020) were obtained directly from the NIS database.

### 2.4. Study Outcomes

The primary outcomes of the study were (a) inpatient mortality rate and (b) healthcare resource utilization, which included length of stay (LoS), total charges, and trends over the study period. The secondary outcomes focused on the trends of comorbidities and malignancies associated with primary sclerosing cholangitis (PSC).

### 2.5. Statistical Analysis

We conducted statistical analyses using STATA version 16.0 (StataCorp, College Station, TX, USA). The NIS’s complex sampling design, which includes stratification, clustering, and weighting, enables the software to produce nationally representative and unbiased results, variance estimates, and *p* values. Patient-level observations were weighted to generalize the findings to the entire U.S. population of hospitalized patients with PSC. Univariate logistic regression was used to calculate unadjusted odds ratios for the primary and secondary outcomes, while multivariate logistic regression adjusted for potential confounders. A forward stepwise model incorporated all confounders significantly associated with the outcomes in the univariate analysis, using a cutoff *p* value of 0.2. Fisher’s exact test compared proportions, and continuous variables were compared using Student’s *t*-test. Two-sided *p* values less than 0.05 were considered to be statistically significant.

To evaluate potential bias from missing data, we assumed the data were not missing at random and applied the multivariate imputation by chained equations (MICE) method. This approach generated multiple imputed datasets using information from all covariates in the database, as well as other covariates used in regression models without missing information. The results with and without imputation did not differ significantly; therefore, results without imputation are reported.

## 3. Results

### 3.1. Trends in PSC-Related Discharges

**Figure 1:**
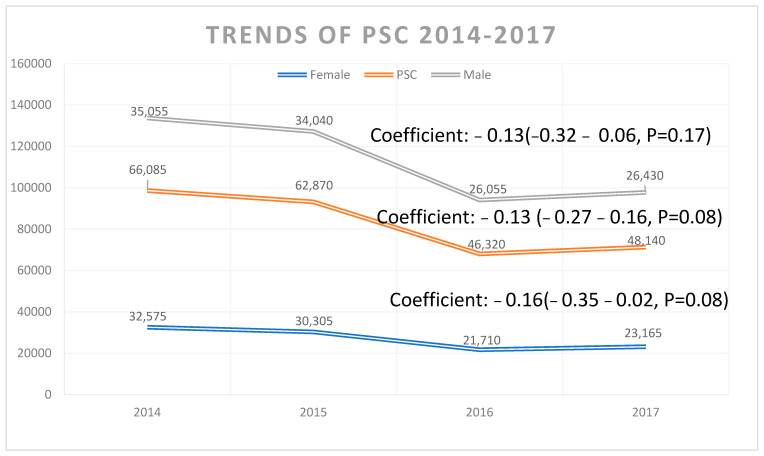
Trends of primary sclerosing cholangitis 2014–2017.

**Figure 2:**
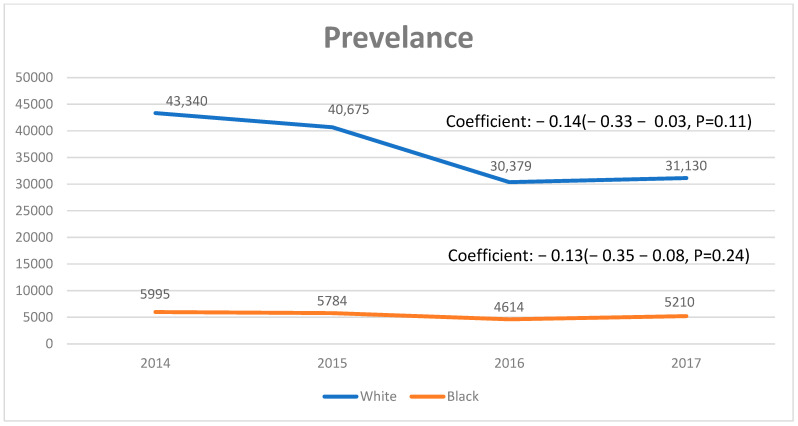
Racial disparity in prevalence of primary sclerosing cholangitis 2014–2017.

Between 2008 and 2017, there were 563,883 discharges with PSC as the primary or secondary diagnosis reported in our study cohort. Overall, there were 100,876 discharges with PSC as a primary diagnosis. The PSC discharge rate per 100,000 people consistently decreased from 2.5 in 2008 to 1.9 in 2017, with an average annual decrease of 0.23% (95% confidence interval [CI]: 0.22–0.25%, *p* < 0.01). In males, the hospital admission increased from 50.8% in 2008 to 53.3% in 2017 (coeff: 0.10; 95% CI: 0.05–0.13, *p* < 0.01), whereas the admissions decreased in females (coefficient: −0.09; 95% CI: −0.13–−0.05%, *p* < 0.01) (Figure 1 and Table 1). There was higher prevalence of PSC in blacks compared to whites (coefficient: 0.11; 95% CI: 0.01–0.06, *p* < 0.02) (Figure 2).

### 3.2. Characteristics of Patients with PSC

The baseline characteristics of patients diagnosed with PSC are summarized in Table 1. Most of these patients were older, with a mean age of 63 years; 63.7% were Caucasian, 52.3% were male, and 54.8% had Medicare as their primary payer (Table 1). The largest subgroup consisted of patients aged from 65 to 85 years, comprising 42.04% of the cohort. Table 2 presents the clinical characteristics of PSC discharges, comparing weighted data from 2008 and 2017. Among these PSC discharges, the percentage of patients with decompensated cirrhosis rose from 14.2% in 2008 to 22.3% in 2017 (coefficient: 0.45, 95% CI: 0.37–0.52). Similarly, cases of compensated hepatic cirrhosis increased from 6.6% to 10.9% (coefficient: 0.44, 95% CI: 0.34–0.55). The proportion of patients with HCC grew from 1.3% to 7.9% (coefficient: 2.13, 95% CI: 1.9–2.8), and the incidence of liver transplants went up from 0.9% to 1.4% (coefficient: 0.46, 95% CI: 1.9–2.8). On the contrary, CCA significantly decreased from 5.1% to 2.8% (coefficient: −0.36, 95% CI: −0.25–−0.46). Overall, the most common complications seen were secondary cholangitis, which accounted for 17.9%, pancreatitis, accounting for 17.4%, and CBD stones with or without a GB stone, accounting for 16.9% (Table 2).

**Figure 3:**
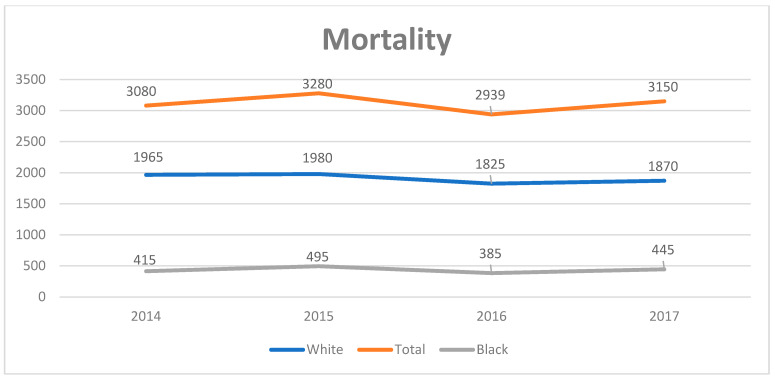
Racial disparity in mortality of primary sclerosing cholangitis 2014–2017.

**Figure 4:**
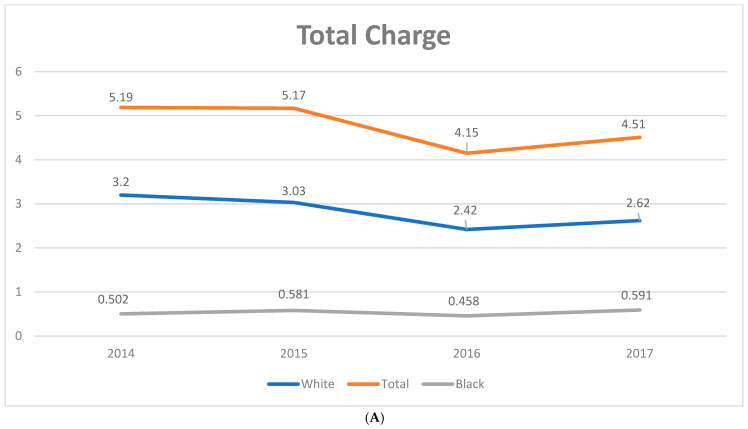

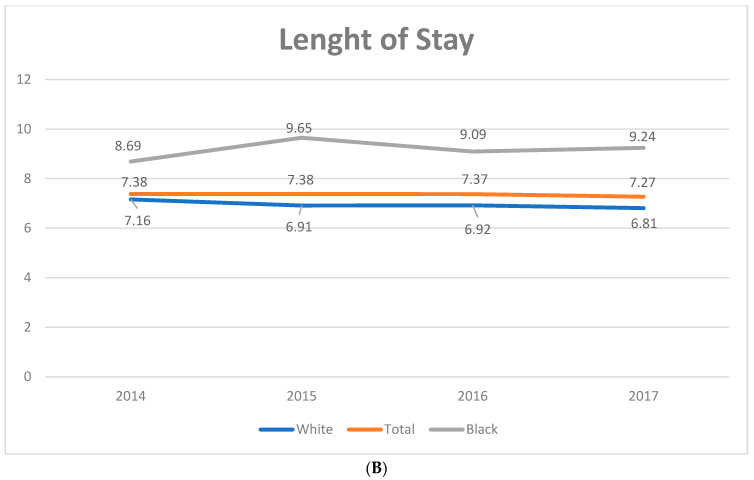
(**A**): Racial disparity in total charge of primary sclerosing cholangitis 2014–2017. (**B**): Racial disparity in length of stay in primary sclerosing cholangitis 2014–2017.

The average total charge increased from USD 61,873 ± 2567 in 2008 to USD 91,262 ± 2961 in 2017, with a significant annual increase by a coefficient of USD 3185 (95% CI: 2502–3867). There was an overall increase in PSC related mortality during the study period by 2.2% ± 0.72, with increase in mortality in African Americans by 6.7% ± 1.3 (Figure 3). In contrast, the average LoS trended down from 8.07 ± 0.18 days in 2008 to 7.27 ± 0.13 days in 2017 (Table 1, Figure 4). The percentage of elective admissions also declined (coefficient: −0.28, 95% CI: −0.41 to −0.16, *p* < 0.01). Around 76% of patients were discharged to their homes (routine: 59.5%; home healthcare: 16.9%), while 13% required transfer to skilled nursing or intermediate care facilities. There was a significant increase in the Medicaid-insured population and a decrease in the uninsured population.

From 2008 to 2017, the APR-DRG severity of illness and risk of death saw significant increases, with major or extreme cases rising from 73.6% to 82.7% (coefficient: 0.21, 95% CI: 0.13–0.28, *p* < 0.02) and the risk of death increasing from 45.3% to 60.9% (coefficient: 0.15, 95% CI: 0.08–0.23), respectively. Congruently, the in-hospital mortality rate among PSC discharges increased annually by a coefficient of 0.25 (95% CI: 0.15–0.36) (Table 1). When patients with PSC were evaluated based on age, over the study time period, the mean age of liver transplantation was 43 years (95% CI: 41.7–44.7), and 91.4% ± 0.90 were younger than 60 years of age.

There were decreases in the prevalence of secondary cholangitis (21.4–15.9%; coefficient: −0.33; 95% CI: −0.40–−0.27), pancreatitis (16.9–12.4%; coefficient: −0.20; 95% CI: −0.26–−0.14), and CBD stones with or without a GB stone (18.2–16.7%; coefficient: −0.33; 95% CI: −0.47–−27). In contrast, there were significant increases in patients with ascites, portal hypertension, hepatic encephalopathy, gastroesophageal varices, and intensive care unit admission (Table 2).

Table 3 provides descriptions of the hospitals where PSC patients were admitted, revealing that the majority were hospitalized in large hospitals (64.2%) and teaching hospitals (65.4%). The average charge for these hospitalizations was USD 75,703 ± USD 931, with an average length of stay (LoS) of 7.5 ± 0.05 days. The total length of hospital stays for all patients admitted from 2008–2017 was 4,237,806 days, with a resulting total hospitalization charge of USD 41.6 billion.

### 3.3. Independent Predictors of In-Hospital Mortality

**Figure 5:**
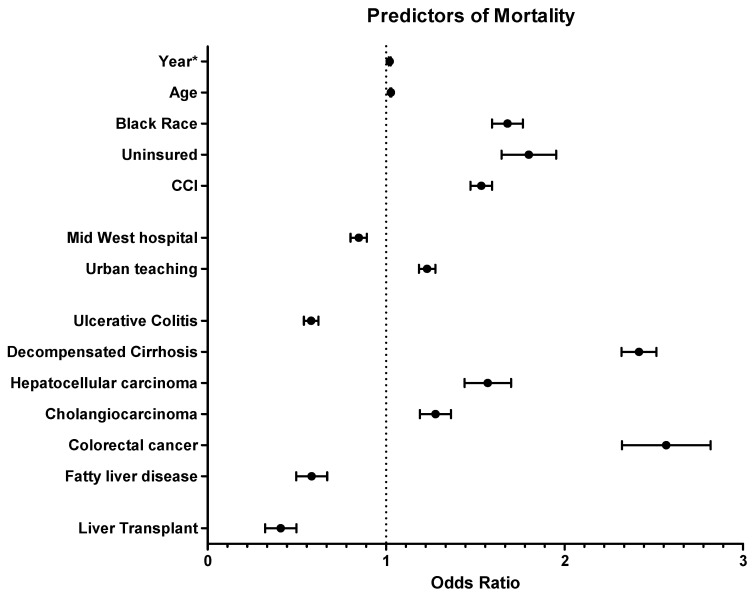
Forest plot for predictors of mortality in primary sclerosing cholangitis. * Year—2008–2017, CCI: Charlson Comorbidity Index.

The upward trend in in-hospital mortality from 2008 to 2017 remained statistically significant, even after adjusting for demographic, clinical, and hospital characteristics (adjusted odds ratio [aOR]: 1.02; 95% CI: 1.01–1.03). Factors independently associated with a higher in-hospital mortality included age (aOR: 1.03; 95% CI: 1.02–1.03), decompensated cirrhosis (aOR: 2.41; 95% CI: 2.25–2.59), CRC (aOR: 2.55; 95% CI: 2.15–3.01), HCC (aOR: 1.56; 95% CI: 1.35–1.80), CCA (aOR: 1.27; 95% CI: 1.13–1.43), and CCI (aOR: 1.53; 95% CI: 1.43–1.64) (Table 4). Additionally, being on Medicaid (aOR: 1.49; 95% CI: 1.12–1.98) or uninsured (aOR: 1.32; 95% CI: 1.17–1.50) compared to Medicare and being Black compared to White (aOR: 1.68; 95% CI: 1.53–1.83) were linked to an increased risk of in-hospital mortality (Table 4, Figure 5).

### 3.4. Independent Predictors of Total Charge

The increasing trend in total charges for PSC discharges remained statistically significant, even after adjusting for demographic, clinical, and hospital characteristics (coefficient: 3742; 95% CI: 3077–4406). The adjusted total charge was found to be increasing by coefficient of USD 3314 (95% CI: 3077–4406) in males, a coefficient of USD 3958 (95% CI: 1737–6178) in patients with decompensated cirrhosis, a coefficient of USD 274,448 (95% CI: 244,522–304,374) in patients with a liver transplant, and USD 4904 (95% CI: 4479–5328) with every point increase in CCI. Additionally, there were variations in charges based on hospital region, size, and location; compared to the Northeast, the West (coefficient: 28,680; 95% CI: 23,054–34,305) had higher charges, and the Midwest (coefficient: −5669; 95% CI: −9991–−1347) had lower charges. In comparison to white patients, African Americans (coefficient: 17,849; 95% CI: 14,203–21,495) and Hispanic patients (coefficient: 1740; 95% CI: 9024–14,455) had higher charges. Compared with non-teaching hospitals, teaching hospitals (coefficient: 1298, 95% CI: 1247–3744) had higher charges (Table 4).

### 3.5. Independent Predictors of LoS

The decrease in length of stay (LoS) among PSC discharges remained statistically significant following adjustments for demographic, clinical, and hospital factors (coefficient: −0.13; 95% CI: −0.09–−0.16%). The adjusted LoS was found to be increasing (coefficient: 1.78, 95% CI: 1.51–2.05) in African Americans compared to white patients and patients with decompensated cirrhosis (coefficient: 1.49, 95% CI: 1.29–1.70), GB cancer (coefficient 0.47, 95% CI: 0.04–0.89), CCA (coefficient: 0.52, 95% CI: 0.28–0.77), CRC (coefficient: 1.44, 95% CI: 1.03–1.93), liver transplant (coefficient of 17.38, 95% CI: 15.20–19.57), and high CCI (Coefficient 0.30, 95% CI: 0.27–0.32). There were also patient-related, hospital region, size, and location variations in the LoS (Table 4).

## 4. Discussion

This study is the largest investigation into the PSC patient population in the United States to date, with data gathered from the NIS from the year 2008 to 2017. The PSC discharge rate per 100,000 people decreased from 2.5 in 2008 to 1.9 in 2017, while the in-hospital mortality rate among PSC discharges increased by 2.3% ± 0.5 (95% CI: 0.15–0.36, *p* < 0.01) throughout the same period. Among PSC discharges, the percentage of patients with decompensated cirrhosis, HCC, and those undergoing liver transplantation increased by 8.3% ± 0.4, 6.6% ± 0.2, and 0.5% ± 0.03, respectively, whereas the percentage of patients with CCA significantly decreased by 2.3% ± 0.02 from 2008 to 2017. We noted that African American patients with a lower socioeconomic status and those that were uninsured were more likely to die in the hospital. 

PSC is a rare clinical entity with limited epidemiological information available. The prevalence of PSC is believed to be around 1 per 10,000 people in northern Europe and from approximately 0.1 to 1.6 per 10,000 people in the United States [3,7]. Studies from northern Europe suggest a rising prevalence of PSC [7,8]. There are several small clinical studies in the existing English literature reporting the prevalence of PSC in certain patient populations. In a UK Clinical-Practice-Research-Datalink-based retrospective cohort study, the prevalence of PSC cases increased from 3.23 per 100,000 patients in 1998 to 7.40 per 100,000 patients in 2014 [16,17]. Another retrospective study from Finland reported the point prevalence of PSC in 2015 to be approximately 31.7 per 100,000 people [18]. In the US, there has been a lack of quality epidemiological studies looking into the prevalence of PSC. In a retrospective study based on the predominantly white population of Olmstead County, Minnesota, the researchers found a prevalence of PSC in the year 2000 of 13.6 per 100,000 persons [19]. Lindkvist et al. performed a retrospective analysis of a population sample from southern Sweden and reported a point prevalence of 16.2 per 100,000 persons in 2005 [20]. In a well-coordinated and massive observational Dutch study published in 2013, Boonstra et al. reported that the prevalence of PSC was 6 per 100,000 patients in 2000, which significantly trended up in 2008 [21]. Based on the limited data available, it may be inferred that the prevalence of PSC is low, but is slowly rising. Interestingly, we delineated an overall decrease in hospital discharges for patients with PSC in our study from 2008–2017. This may be due to a higher selectivity in admissions and an emphasis on outpatient care, despite the progressive disease course of PSC.

From 2008 to 2017, we noted a significant increase in patients with both compensated and decompensated cirrhosis and associated complications such as portal hypertension, ascites, hepatic encephalopathy, gastroesophageal varices, and liver transplantation. This was expected given the natural course of PSC [17,22]. Concomitantly with these findings, we found a six-fold increase in the development of HCC from 1.3% to 7.9% (coefficient of 2.13, 95% CI: 1.9–2.8), which may suggest that increased HCC screening for patients with non-cirrhotic PSC may be warranted outside of the current screening guidelines for HCC.

The most devastating complication of PSC is CCA. The median time to develop CCA is from around 2 to 6 years after a diagnosis of PSC [22]. About 10% of patients with PSC may eventually develop CCA. About 80% of patients diagnosed with CCA die within a year of diagnosis [21]. Based on our analysis, the inpatient deaths associated with CCA decreased from 2008 to 2017. In 2008, Charatcharoenwitthaya et al. prospectively surveyed the utility of screening patients with PSC for cholangiogram using CA 19-9 and a cross-sectional imaging technique. They reported that the incidence of CCA in patients with PSC was 1.2%. Approximately two-thirds of cancers were detected at an early stage of disease, and the majority were treated with a liver transplant. The American College of Gastroenterology recommends screening all PSC patients with MRCP and CA19-9 once every 6–12 months [6,23,24]. If diagnosed early, CCA may be definitively treated with surgical resection or liver transplantation [25]. Interestingly, in our study, we noticed that the number of patients with PSC undergoing liver transplantation increased from 2008 to 2017. Patients with PSC may also develop other hepatobiliary malignancies, colon cancer, bacterial cholangitis, and cirrhosis. Patients with advanced PSC or PSC complicated with CCA or HCC often require liver transplantation for the management of advanced disease. Hence, patients with PSC are at risk for recurrent hospitalization during their lifetime [26]. However, in this investigation, we noted that the number of inpatient admissions trended down from 2008 to 2017.

We observed that African American patients were more likely to have poor outcomes as compared to the white population. Both disease prevalence and mortality in this population increased over the study period. Other disadvantaged populations, such as those that are uninsured and those on Medicaid, also had an increased inpatient mortality, which suggests that there is a socioeconomic component regarding poorer outcomes related to PSC. Bowlus et al. performed an observational analysis of the liver transplant registrants of the United Network for Organ Sharing (UNOS) Network. It was found that African Americans experienced PSC at rates comparable to those of white people; however, they tended to be younger and have higher Model for End-Stage Liver Disease (MELD) scores at the time of liver transplantation registration compared to white and Hispanic people, indicating a more severe disease phenotype [27]. Similarly, in another United-Network-for-Organ-Sharing-based study, Wilder at al. determined that young African Americans with PSC listed for liver transplant had a higher wait-list mortality, acute rejection, graft failure, and post-liver-transplant 5-year mortality [28]. Based on the cumulative evidence in the existing English literature, we believe that these racial disparities in the outcomes of patients with PSC must be further investigated, so that earlier interventions in this population may become possible.

Our analysis benefits from the extensive size of the patient cohort and the duration of our study period. This study represents the first research on a nationwide sample of hospitalized PSC cases in the United States from 2008 to 2017, making it the largest inpatient database to document national trends.

### Limitations

There are some limitations of this study. The use of ICD codes for identifying PSC patients precluded the clinical confirmation of disease status. Nonetheless, the definition of PSC is standardized and widely accepted [29]. Additionally, due to the design of the NIS database, readmissions are not accounted for, potentially leading to an underestimation of procedural complication rates. The study period (2008–2017) was chosen due to the relevance and consistency of the data, which capture significant trends and policies within this timeframe, where the ICD codes remained uniform and homogenous. Extending beyond 2017 would introduce variables that could complicate the analysis and reduce consistency. While incorporating current data could be insightful, it would require a different scope, better suited for future research.

We described the risk factors associated with increased odds of mortality. However, due to the relatively small sample size of mortality cases in our database, we were unable to perform a detailed analysis of the specific causes of death or to assess differences between racial groups. The low number of mortality events limits the statistical power required to reliably determine and compare causes of death. We acknowledge the importance of understanding cause-specific mortality and suggest that future research with larger sample sizes would be beneficial to explore these aspects in more detail. Due to the small number of interventional endoscopic procedures performed within our study population, we were unable to provide a detailed analysis or accurate reporting on these procedures. Consequently, the data on interventional procedures during admissions were also limited. Our research showed increased odds of mortality in uninsured patients, and as such, we did not investigate temporal changes in uninsurance status or its impact on disease complications. This is a potential area for further investigation and research.

Despite its limitations, the results from this large database study demonstrated rising mortality, healthcare resource utilization, and racial disparity associated with PSC in the United States. Hepatologists should be cognizant of these trends and follow up with closer cancer surveillance in these patients.

## Figures and Tables

**Table 1:** Characteristics of PSC discharges, NIS 2008–2017.

Values Listed as Mean (95% CI) or % (N/Total) Unless Otherwise Listed	2008–2017 (Weighted)	2008 (Weighted)	2017 (Weighted)	Coefficient (95% CI)	*p*-Value
**Age, years**	63.29 ± 0.37	63.29 ± 0.70	62.15 ± 0.37	−0.14 (−0.27–−0.01)	0.030
**Race, %**					
White	63.73 ± 0.53	58.09 ± 2.11	64.02 ± 1.14	Reference	
Black	9.06 ± 0.18	6.36 ± 0.55	11.05 ± 0.49	0.23 (0.10–0.37)	<0.01
Hispanic	10.81 ± 0.27	8.98 ± 0.96	11.56 ± 0.58	0.07 (−0.10–0.23)	0.425
Other ^†^	16.41 ± 0.48	26.67 ± 2.15	13.36 ± 1.15	−0.61 (−0.82–−0.40)	<0.01
Male, %	52.30 ± 0.19	50.85 ± 0.73	53.29 ± 0.56	0.10 (0.05–0.13)	<0.01
**Primary Payer, %**					
Medicare	54.82 ± 0.31	52.69 ± 1.28	53.26 ± 0.69	Reference	
Medicaid	10.39 ± 0.18	9.63 ± 0.75	12.09 ± 0.44	0.20 (0.08–0.33)	0.002
Private	28.40 ± 0.28	31.63 ± 1.04	29.44 ± 0.72	−0.06 (−0.14–0.03)	0.210
Uninsured	6.39 ± 1.38	6.06 ± 0.44	5.21 ± 0.28	−0.27 (−0.39–−0.14)	<0.01
Elective admission, %	9.33 ± 0.25	10.62 ± 0.73	8.06 ± 0.41	−0.28 (−0.41–−0.16)	<0.01
**Disposition status, %**					
Routine	59.55 ± 0.46	60.11 ± 0.84	54.89 ± 0.67	Reference	
Short-term hospital	4.69 ± 0.08	4.74 ± 0.33	5.15 ± 0.27	0.11 (−0.03–0.24)	0.128
SNF/ICF/Other	13.33 ± 0.15	13.88 ± 0.57	13.98 ± 0.42	0.07 (−0.02–0.16)	0.121
Home healthcare	16.92 ± 0.19	15.55 ± 0.65	18.88 ± 0.48	0.24 (0.15–0.33)	<0.01
Against medical advice	0.36 ± 0.02	0.51 ± 0.07	0.69 ± 0.08	0.17 (−0.13–0.47)	0.266
**Died ^‡^**	5.15 ± 0.07	5.21 ± 0.24	6.41 ± 0.25	0.25 (0.15–0.36)	<0.01
Resource utilization					
Number of procedures					
**LoS, days**	7.52 ± 0.05	8.07 ± 0.18	7.27 ± 0.13	−0.08 (−0.12–−0.04)	<0.01
**Charge, $ ^§^**	75,703 ± 931	61,873 ± 2567	91,262 ± 2961	3185 (2502–3867)	<0.01
**Cost, USD ^§||^**	22,007 ± 234	21,338 ± 695	23,330 ± 757	242 (78–407)	0.004

Data presented as weighted mean/percentage ± standard error. Statistically significant *p* value was considered as *p* < 0.05. ^†^ Other races include Asian/Native American; 7.7% of race was missing. ^‡^ In-hospital mortality. § Inflation adjusted to 2017 dollars. ^||^ Cost was converted from charges using the HCUP cost-to-charge ratio. Abbreviations: ICF, intermediate care facility; and SNF, skilled nursing facility. Abbreviations: ICF, intermediate care facility; and SNF, skilled nursing facility.

**Table 2:** Clinical characteristics of PSC discharges, NIS 2008–2017.

Values Listed as Mean (95% CI) or % (N/Total) Unless Otherwise Listed	2008–2017 (Weighted)	2008 (Weighted)	2017 (Weighted)	Coefficient (95% CI)	*p* Value
Number diagnosed					
**Severity of illness, %**					
Minor/moderate loss of function	16.36 ± 0.19	26.44 ± 0.52	17.25 ± 0.42	Reference	
Major/extreme loss of function	83.64 ± 0.19	73.56 ± 0.52	82.75 ± 0.42	0.21 (0.13–0.28)	<0.01
**Risk of dying, %**					
Minor/moderate loss of function	36.44 ± 0.37	54.73 ± 0.66	39.07 ± 0.52	Reference	
Major/extreme loss of function	63.56 ± 0.37	45.27 ± 0.66	60.93 ± 0.53	0.15 (0.08–0.23)	<0.01
**Severity of Cirrhosis**					
Compensated cirrhosis, %	8.35 ± 0.13	6.63 ± 0.49	10.91 ± 0.46	0.44 (0.34–0.55)	<0.01
Primary biliary cirrhosis, %	0.97 ± 0.04	1.11 ± 0.17	0.60 ± 0.08	−0.42 (−0.21–−0.62)	<0.01
Decompensated cirrhosis, %	18.27 ± 0.18	14.18 ± 0.70	22.33 ± 0.55	0.45 (0.37–0.52)	<0.01
**Coexisting malignancy**					
Hepatocellular carcinoma, %	2.38 ± 0.07	1.37 ± 0.17	7.92 ± 0.39	2.13 (1.96–2.84)	<0.01
Gall bladder carcinoma, %	0.89 ± 0.03	0.74 ± 0.09	1.13 ± 0.11	0.28 (0.09–0.48)	0.004
Cholangiocarcinoma, %	4.96 ± 0.10	5.12 ± 0.35	2.79 ± 0.17	−0.36 (−0.25–−0.46)	<0.01
Colorectal cancer, %	1.26 ± 0.04	1.15 ± 0.13	1.58 ± 0.12	0.34 (0.17–0.52)	<0.01
**Overlap with autoimmune diseases**					
Inflammatory bowel disease, %	6.77 ± 0.13	6.30 ± 0.53	8.11 ± 0.44	0.22 (0.09–0.35)	0.001
Ulcerative colitis, %	4.69 ± 0.11	4.36 ± 0.47	5.66 ± 0.36	0.22 (0.07–0.38)	0.004
Autoimmune hepatitis, %	1.11 ± 0.04	0.37 ± 0.07	1.94 ± 0.17	0.84 (0.61–1.06)	<0.01
Autoimmune disease, % ^†^	0.22 ± 0.01	0.27 ± 0.05	0.33 ± 0.06	0.13 (−0.26–0.52)	0.510
Fatty liver disease	2.61 ± 0.05	1.41 ± 0.11	3.11 ± 2.76	0.55 (0.43–0.66)	<0.01
Clostridium difficile	2.15 ± 0.05	2.09 ± 0.16	1.98 ± 0.14	0.03 (−0.10–0.16)	0.650
Marijuana use	0.47 ± 0.02	0.17 ± 0.04	0.84 ± 0.09	1.18 (1.05–1.42)	<0.01
Liver transplant, %	0.86 ± 0.05	0.86 ± 0.17	1.42 ± 0.17	0.46 (0.06–0.85)	0.022
**CCI**	2.34 ± 0.01	2.13 ± 0.04	3.12 ± 0.03		
Secondary cholangitis	17.88 ± 0.16	21.39 ± 0.66	15.91 ± 0.43	−0.33 (−0.40–−0.27)	<0.01
CBD stone with or without GB stone	15.97 ± 0.19	18.21 ± 0.82	16.75 ± 0.43	−0.33 (−0.47–−27)	<0.01
Mechanical complication of bile duct prosthesis	2.81 ± 0.06	3.04 ± 0.22	1.86 ± 0.15	−0.40 (–0.53–−0.28)	<0.01
Pancreatitis	17.42 ± 0.15	16.93 ± 0.51	12.44 ± 0.38	−0.20 (−0.26–−0.14)	<0.01
**Complications, %**					
Ascites	8.21 ± 0.01	6.84 ± 0.46	10.99 ± 0.42	0.46 (0.36–0.55)	<0.01
Hepatic encephalopathy	3.82 ± 0.08	2.06 ± 0.21	9.60 ± 0.38	1.46 (1.32–1.59)	<0.01
Portal hypertension	4.40 ± 0.10	3.00 ± 0.29	7.11 ± 0.38	0.73 (0.58–0.88)	<0.01
Gastroesophageal varices	3.07 ± 0.08	2.43 ± 0.24	4.06 ± 0.27	0.43 (0.28–0.58)	<0.01
ICU admission	6.87 ± 0.09	6.18 ± 0.29	7.29 ± 0.29	0.12 (0.05–0.20)	0.002

Data presented as mean/percentage ± standard error. Statistically significant *p* value was considered as *p* < 0.05. ^†^ Autoimmune disease scleroderma, Sjögren’s syndrome, and systemic sclerosis.

**Table 3:** Characteristics of hospitals, 2008–2017.

Characteristics	2008–2017 (Weighted)	2008 (Weighted)	2017 (Weighted)	Coefficient (95% CI)	*p* Value
**Hospital Region, %**					
Northeast	21.89 ± 0.67	23.60 ± 3.50	20.95 ± 1.59	Reference	
Midwest	22.35 ± 0.68	19.98 ± 2.52	22.89 ± 1.75	0.07 (−0.22–0.37)	0.614
South	31.23 ± 0.63	29.31 ± 2.68	32.60 ± 1.69	0.14 (−0.11–0.38)	0.266
West	24.51 ± 0.63	27.10 ± 3.04	23.54 ± 1.48	−0.01 (−0.29–0.27)	0.961
**Hospital bed size, %**					
Small	11.98 ± 0.29	8.44 ± 0.95	14.80 ± 0.88	Reference	
Medium	23.84 ± 0.48	22.33 ± 2.10	24.49 ± 1.33	−0.34 (−0.51–−0.16)	<0.01
Large	64.18 ± 0.60	69.22 ± 2.47	60.71 ± 1.65	−0.65 (−0.83–−0.47)	<0.01
**Hospital location/teaching, %**					
Rural or urban nonteaching	34.60 ± 0.63	44.19 ± 3.16	20.66 ± 1.06	Reference	
Urban teaching	65.40 ± 0.63	55.80 ± 3.16	79.34 ± 1.06	1.04 (1.02–1.20)	<0.01

Data presented as percentage ± standard error. Statistically significant *p* value was considered as *p* < 0.05.

**Table 4:** Predictors of in-hospital mortality, charge, and LoS, NIS 2008–2017.

Characteristics	In-Hospital Mortality * OR (95% CI)	Charge ^‡^ IRR (95% CI)	LoS ^‡^ IRR (95% CI)
Calendar year	1.02 (1.01–1.03)	3742 (3077–4406)	−0.13 (−0.09–−0.16)
Age, years	1.03 (1.02–1.03)	−28 (−83–28)	−0.002 (−0.008–0.004)
**Race**			
White	Reference	Reference	Reference
Black	1.68 (1.53–1.83)	17,849 (14,203–21,495)	1.78 (1.51–2.05)
Hispanic	1.03 (0.93–1.13)	11,740 (9024–14,455)	0.67 (0.44–0.90)
Other ^†^	1.04 (0.94–1.13)	1105 (−1237–3447)	0.41 (0.24–0.59)
Male	1.02 (1.01–1.04)	3314 (2326–4302)	0.39 (0.27–0.50)
**Primary Payer**			
Medicare	Reference	Reference	Reference
Medicaid	1.46 (1.31–1.63)	−3080 (−5825–−335)	0.83 (0.55–1.11)
Private	1.22 (0.88–1.34)	2525 (83–4219)	−0.98 (−1.15–−0.81)
Uninsured	1.79 (1.54–2.07)	−3926 (−6460–−1391)	−0.59 (−0.87–−0.31)
**Hospital Region**			
Northeast	Reference	Reference	Reference
Midwest	0.84 (0.77–0.93)	−5669 (−9991–−1347)	−1.14 (−1.36–−0.91)
South	1.03 (0.95–1.12)	−4043 (−8284–197)	−0.18 (−0.40–0.04)
West	1.02 (0.93–1.12)	28,680 (23,054–34,305)	−0.66 (−0.93–−0.40)
**Hospital bed size**			
Small	Reference	Reference	Reference
Medium	1.05 (0.94–1.31)	1499 (−1091–4088)	0.71 (0.47–0.95)
Large	1.26 (0.94–1.34)	5449 (2829–8068)	1.29 (1.07–1.51)
**Hospital location/teaching**			
Rural/urban nonteaching	Reference	Reference	Reference
Urban teaching	1.23 (1.15–1.31)	1298 (1247–3744)	1.33 (1.18–1.48)
Ulcerative colitis	0.61 (0.50–0.63)	−7577 (−10,155–−4998)	−1.90 (−2.18–−1.62)
**Comorbidities**			
Decompensated cirrhosis	2.41 (2.25–2.59)	3958 (1737–6178)	1.49 (1.29–1.70)
Hepatocellular carcinoma	1.56 (1.35–1.80)	−237 (−4944–4471)	−0.04 (−0.38–0.31)
Gall bladder carcinoma	1.11 (0.87–1.42)	5095 (−2249–12,439)	0.47 (0.04–0.89)
Cholangiocarcinoma	1.27 (1.13–1.43)	2279 (−913–5470)	0.52 (0.28–0.77)
Colorectal cancer	2.55 (2.15–3.01)	3613 (−2130–9357)	1.44 (1.03–1.93)
Autoimmune hepatitis	1.34 (0.75–1.35)	238 (−8407–8884)	−1.68 (−2.13–−1.23)
Liver transplant	0.39 (0.27–0.57)	274,448 (244,522–304,374)	17.38 (15.20–19.57)
Fatty liver	0.57 (0.44–0.74)	−430 (−2652–1792)	−0.37 (−0.61–−0.13)
Marijuana use	0.93 (0.54–1.56)	−2383 (−8076–3309)	−0.87 (−1.49–−0.25)
ICU admission	16.16 (14.98–17.42)	59,501 (53,430–65,572)	10.26 (9.76–10.76)
CCI > 1	1.53 (1.43–1.64)	4904 (4479–5328)	0.30 (0.27–0.32)
LoS, days	1.03 (1.02–1.04)	10,158 (9518–10,799)	NA

Note: Due to multicollinearity, complications and cholangitis were excluded in the models. Inflation-adjusted charges to 2017 dollars. Bold text denotes *p* < 0.05. * Analyzed by multilevel logistic regression. ^‡^ Analyzed by multilevel generalized Poisson model. ^†^ Other races include Asian/Native American, LoS: Length of stay. Abbreviations: NA, not applicable; OR, odds ratio; and RR, incidence rate ratio.

## Data Availability

Data is available in the supplementary file, incase of any further questions corresponding author can be directly con-tacted to provide more data.

## Supplementary Materials

The following supporting information can be downloaded at: https://www.mdpi.com/article/10.3390/diagnostics14222493/s1, Table S1: ICD-9-CM and ICD-10-CM diagnosis Codes Used for Data Extraction and Analysis from the Nationwide Inpatient sample Database 2011–2017.

## References

[1] Karlsen T.H., Folseraas T., Thorburn D., Vesterhus M. (2017). Primary sclerosing cholangitis—A comprehensive review. J. Hepatol..

[2] Carrijo I., Ugrinovich L., Simioni P. (2019). Primary sclerosing cholangitis associated with inflammatory bowel disease. Int. Med..

[3] Hirschfield G.M., Karlsen T.H., Lindor K.D., Adams D.H. (2013). Primary sclerosing cholangitis. Lancet.

[4] Lazaridis K.N., Larusso N.F. (2016). Primary Sclerosing Cholangitis. New Engl. J. Med..

[5] Palmela C., Peerani F., Castaneda D., Torres J., Itzkowitz S.H. (2018). Inflammatory Bowel Disease and Primary Sclerosing Cholangitis: A Review of the Phenotype and Associated Specific Features. Gut Liver.

[6] Lindor K.D., Kowdley K.V., Harrison E.M. (2015). American College of Gastroenterology. ACG Clinical Guideline: Primary sclerosing cholangitis. Am. J. Gastroenterol..

[7] Boonstra K., Beuers U., Ponsioen C.Y. (2012). Epidemiology of primary sclerosing cholangitis and primary biliary cirrhosis: A systematic review. J. Hepatol..

[8] Molodecky N.A., Kareemi H., Parab R., Barkema H.W., Quan H., Myers R.P., Kaplan G.G. (2011). Incidence of primary sclerosing cholangitis: A systematic review and metaanalysis. Hepatology.

[9] Overview of the National (Nationwide) Inpatient Sample (NIS) In NIS Overview; Agency for Healthcare Research and Quality, Rockville, USA: 2019. https://www.hcup-us.ahrq.gov/nisoverview.jsp.

[10] Rockville M. (2002). The healthcare cost and utilization project: An overview. Eff. Clin. Pract..

[11] 2016 Introduction to the NIS In Healthcare Cost and Utilization Project (HCUP); Agency for Healthcare Research and Quality Rockville, USA: 2018. www.hcup-us.ahrq.gov/db/nation/nis/NIS_Introduction_2016.jsp.

[12] Technical Supplement 13. In Comparative Analysis of HCUP and NHDS Inpatient Discharge Data; Agency for Healthcare Research and Quality:, Rockville, USA 2012. http://www.ahrq.gov/research/data/hcup/nhds/niscomp.html.

[13] HCUP Quality Control Procedures In Healthcare Cost and Utilization Project; Agency for Healthcare Research and Quality: Rockville, USA 2019. https://www.hcup-us.ahrq.gov/db/quality.pdf.

[14] Quan H., Sundararajan V., Halfon P., Fong A., Burnand B., Luthi J.-C., Saunders L.D., Beck C.A., Feasby T.E., Ghali W.A. (2005). Coding algorithms for defining comorbidities in ICD-9-CM and ICD-10 administrative data. Med. Care.

[15] McCormick P.J., Lin H.M., Deiner S.G., Levin M.A. (2018). Validation of the All Patient Refined Diagnosis Related Group (APR-DRG) Risk of Mortality and Severity of Illness Modifiers as a Measure of Perioperative Risk. J. Med. Syst..

[16] White I.R., Royston P., Wood A.M. (2010). Multiple imputation using chained equations: Issues and guidance for practice. Stat. Med..

[17] Liang H., Manne S., Shick J., Lissoos T., Dolin P. (2017). Incidence, prevalence, and natural history of primary sclerosing cholangitis in the United Kingdom. Medicine.

[18] Barner-Rasmussen N., Pukkala E., Jussila A., Färkkilä M. (2020). Epidemiology, risk of malignancy and patient survival in primary sclerosing cholangitis: A population-based study in Finland. Scand. J. Gastroenterol..

[19] Bambha K., Kim W., Talwalkar J., Torgerson H., Benson J.T., Therneau T.M., Loftus E.V., Yawn B.P., Dickson E.R., Melton L.J. (2003). Incidence, clinical spectrum, and outcomes of primary sclerosing cholangitis in a United States community. Gastroenterology.

[20] Kaplan G.G., Laupland K.B., Butzner D., Urbanski S.J., Lee S.S. (2007). The Burden of Large and Small Duct Primary Sclerosing Cholangitis in Adults and Children: A Population-Based Analysis. Am. J. Gastroenterol..

[21] Boonstra K., Weersma R.K., van Erpecum K.J., Rauws E.A., Spanier B.W., Poen A.C., van Nieuwkerk K.M., Drenth J.P., Witteman B.J., Tuynman H.A. (2013). Population based epidemiology, malignancy risk, and outcome of primary sclerosing cholangitis. Hepatology.

[22] Takakura W.R., Tabibian J.H., Bowlus C.L. (2017). The evolution of natural history of primary sclerosing cholangitis. Curr. Opin. Gastroenterol..

[23] Charatcharoenwitthaya P., Enders F.B., Halling K.C., Lindor K.D. (2008). Utility of serum tumor markers, imaging, and biliary cytology for detecting CCA in primary sclerosing cholangitis. Hepatology.

[24] Razumilava N., Gores G.J., Lindor K.D. (2011). Cancer surveillance in patients with primary sclerosing cholangitis. Hepatology.

[25] Chapman R., Fevery J., Kalloo A., Nagorney D.M., Boberg K.M., Shneider B., Gores G.J. (2010). Diagnosis and management of primary sclerosing cholangitis. Hepatology.

[26] Merion R.M., Shearon T.H., Berg C.L., Everhart J.E., Abecassis M.M., Shaked A., Fisher R.A., Trotter J.F., Brown R.S., Terrault N.A. (2010). Hospitalization Rates Before and After Adult-to-Adult Living Donor or Deceased Donor Liver Transplantation. Ann. Surg..

[27] Bowlus C.L., Li C.-S., Karlsen T.H., Lie B.A., Selmi C. (2010). Primary sclerosing cholangitis in genetically diverse populations listed for liver transplantation: Unique clinical and human leukocyte antigen associations. Liver Transplant..

[28] Wilder J., Henson J., Patel Y., Zheng J., Chow S.-C., Muir A. (2016). Racial Differences in Phenotype and Outcome Among Patients with Primary Sclerosing Cholangitis Listed for Liver Transplant: 2016 ACG Fellows-In-Training Award (Liver Category). Am. J. Gastroenterol..

[29] Wiencke K., Boberg K. (2011). Current consensus on the management of primary sclerosing cholangitis. Clin. Res. Hepatol. Gastroenterol..

