# Supplementary material for: Racial Disparities in Inpatient Hospital Outcomes of Primary Sclerosing Cholangitis in United States: Nationwide Analysis
Source: Diagnostics (Basel). 2024 Nov 7;14(22):2493. doi: 10.3390/diagnostics14222493 (PMC11592423; doi:10.3390/diagnostics14222493)
Supplement: Supplementary file 1 [file diagnostics-14-02493-s001.zip › diagnostics-3105867-supplementary.pdf]

diagnostics

Supplementary Materials

Table S1. ICD-9-CM and ICD-10-CM diagnosis Codes Used for Data Extraction and Analysis from the Nationwide Inpatient sample Database 2011-2017.

Variables	ICD-9- CM Code	ICD-10- CM Code
Primary Sclerosing Cholangitis	576.1	K83.01, K83.09
Compensated Cirrhosis	571.2, 571.5	K70.30, K74.0, K74.60, K74.69, K74.4, K74.5
Decompensated Cirrhosis	456.0, 456.2, 572.2, 572.3, 572.4, 782.4 789.5, 782.4, 348.3, 789.5, 789.59, 567.23 567.0, 567.2, 567.8, 567.9, 456.0, 456.1, 456.2, 456.20, 456.21, 572.3, 572.2, 572.4, 573.5, 452	I85.0, I86.4, I98.20, I98.3, K72.1, K72.9, K76.6, K76.7
Primary biliary cirrhosis	571.6	K74.3
Hepatocellular carcinoma	155.0	C22xx
Gall bladder cancer	156.0	C23
Cholangiocarcinoma	155.1, 156.1, 156.9, 156.8	C24xx
Colorectal cancer	153xx, 154xx	C20xx, C18xx
Ulcerative colitis	556x	K51xx
Autoimmune hepatitis	571.42	K75.4
Nonalcoholic fatty liver disease	571.5	K76.0
Clostridium infection	008.45	A04.71, A04.72
Marijuana	305.2	F12xx
Liver transplant	50.51, 50.59	0FY00Z0, 0FY00Z1, 0FY00Z2
Pancreatitis	577.0	K85.00, K85.01, K85.02, K85.10, K85.11, K85.12, K85.20, K85.21, K85.22, K85.30, K85.31, K85.32, K85.80, K85.81, K85.82, K85.90, K85.91, K85.92
Common bile duct stone	57.40, 57.41, 57.42, 57.43, 57.44, 57.45	K803xx, K804xx, K805xx, K806xx, K807xx, K808xx
Poral Hypertension	572.3	K76.6
Ascites	789.5, 789.59, 789.51	R18.8, R18.0
Hepatic Encephalopathy	572.2	K72xx

Mechanical Ventilation (A)	967, 96.70, 96.71, 96.72	5A1935Z, 5A1945Z, 5A1955Z
Vasopressor use (B)	0017	3E030XZ, 3E033XZ, 3E040XZ, 3E043XZ, 3E050XZ, 3E053XZ, 3E060XZ,3E063XZ

A+B: ICU admission.
